# Comprehensive characterization of cancer‐testis genes in testicular germ cell tumor

**DOI:** 10.1002/cam4.2223

**Published:** 2019-05-09

**Authors:** Yuting Chang, Xuewei Wang, Yide Xu, Liu Yang, Qufei Qian, Sihan Ju, Yao Chen, Shuaizhou Chen, Na Qin, Zijian Ma, Juncheng Dai, Hongxia Ma, Guangfu Jin, Erbao Zhang, Cheng Wang, Zhibin Hu

**Affiliations:** ^1^ Department of Epidemiology, Center for Global Health, School of Public Health Nanjing Medical University Nanjing China; ^2^ Department of Bioinformatics, School of Biomedical Engineering and Informatics Nanjing Medical University Nanjing China; ^3^ State Key Laboratory of Reproductive Medicine Nanjing Medical University Nanjing China; ^4^ Jiangsu Key Lab of Cancer Biomarkers, Prevention and Treatment, Jiangsu Collaborative Innovation Center for Cancer Personalized Medicine Nanjing Medical University Nanjing China

**Keywords:** cancer‐testis gene, stem cell maintenance, survival, testicular germ cell tumor

## Abstract

Cancer‐testis (CT) genes are a group of genes restrictedly expressed in testis and multiple cancers and can serve as candidate driver genes participating in the development of cancers. Our previous study identified a number of CT genes in nongerm cell tumors, but their expression pattern in testicular germ cell tumor (TGCT), a cancer type characterized by less genomic alterations, remained largely unknown. In this study, we systematically investigated the expression pattern of CT genes in TGCT samples and evaluated the transcriptome difference between TGCT and normal testis tissues, using datasets from the UCSC Xena platform, The Cancer Genome Atlas (TCGA) and the Genotype‐Tissue Expression (GTEx) project. Pathway enrichment analysis and survival analysis were conducted to evaluate the biological function and prognostic effect of expressed CT genes. We identified that 1036 testis‐specific expressed protein‐coding genes and 863 testis‐specific expressed long noncoding RNAs (lncRNAs) were expressed in TGCT samples, including 883 CT protein‐coding genes and 710 CT lncRNAs defined previously. The number of expressed CT genes was significantly higher in seminomas (*P *= 3.48 × 10^−13^) which were characterized by frequent mutations in driver genes (*KIT*, *KRAS* and *NRAS*). In contrast, the number of expressed CT genes showed a moderate negative correlation with the fraction of copy number altered genomes (cor = −0.28, *P *= 1.20 × 10^−3^). Unlike other cancers, our analysis revealed that 96.16% of the CT genes were down‐regulated in TGCT samples, while CT genes in stem cell maintenance related pathways were up‐regulated. Further survival analysis provided evidence that CT genes could also predict the prognosis of TGCT patients with both disease‐free interval and progression‐free interval as clinical endpoints. Taken together, our study provided a global view of CT genes in TGCT and provided evidence that CT genes played important roles in the progression and maintenance of TGCT.

## 1 INTRODUCTION

Cancer‐testis (CT) genes are predominantly expressed in normal testis tissues and a wide type of cancers.^1^ They were first named as Cancer‐testis Antigens (CTAs) since early defined CT genes can encode immunogenic antigens which evoke immune responses in cancer patients.^2^, ^3^ Because of the immunogenicity and restricted expression pattern in normal tissues, CTAs are commonly considered as ideal immunotherapy targets for cancer therapy.^3^, ^4^, ^5^ Recently, CT genes have also been proved that contribute to various neoplastic phenotype features,^3^ and can be driver candidates participating in the process of tumorigenesis.^6^ In our recent work, we performed a systematic identification of CT genes by integrating gene expression data of 24 normal tissues and tumor samples from 19 cancer types and defined 876 new CT genes.^6^ However, all previous studies investigated CT genes in nongerm cell tumors, leaving a blank for testicular germ cell tumor (TGCT).

Testicular cancer is the most commonly diagnosed malignancy of adult males among Americans,^7^ while TGCT accounts for more than 95% of testicular cancers.^8^ The main types of TGCT are seminomas and non‐seminomas, and non‐seminomas consist of either undifferentiated (embryonal carcinoma) or differentiated (teratoma, yolk sac tumor, and choriocarcinoma) histologic subtypes.^9^ In a recent study, Shen et al identified that TGCTs were characterized by a lack of genomic alterations, deficiency of methylation level and high aneuploidy,^10^ which highlighted the importance of transcriptome alteration in the development of TGCT. Considering the special expression pattern, the transcriptome alteration of CT genes may participate in the development of TGCT. Interestingly, some CT genes have previously been reported to be expressed in TGCT samples and have a potential function in tumorigenesis, such as *CTAG1B* 11 and the MAGE families *MAGEA1*, *MAGEA2*, *MAGEA3,* and *MAGEA4*.^12^, ^13^, ^14^, ^15^ Although attracting more attention, CT genes in the process of TGCT development have not been well characterized.

In this study, to make a comprehensive description of CT genes in TGCT, we first described the expression pattern of CT protein‐coding genes and CT long noncoding RNAs (CT‐lncRNAs) in TGCT samples. Then we conducted differential expression analysis between normal and malignant testis samples to provide a global view of the expression alterations of CT genes between these samples. Additionally, we performed pathway enrichment analysis to investigate the function of these altered CT genes, as well as survival analysis to evaluate the prognostic effect of CT genes. Overall, our study provided evidence that CT genes may play important roles in the progression and maintenance of TGCT.

## 2 METHODS

### 2.1 Quantification expression data of testicular germ cell cancer samples used in this study

To comprehensively explore the expression pattern of our previous defined testis‐specific expressed genes (TSGs)^6^ in TGCT, we retrieved quantification expression data of 150 TGCT samples from the University of California Santa Cruz (UCSC) Xena website (https://xenabrowser.net/datapages/).^16^ The expression data were quantified as raw read counts. In our previous study,^6^ we analyzed the transcriptome data of normal tissues (24 different organs from 175 individuals) from the Genotype‐Tissue Expression (GTEx) project (https://www.gtexportal.org/home/) and used specificity measure to classify all human genes (50 016) into six categories (C1‐C6) based on their expression pattern, including high confidence testis‐specific expressed protein coding genes (C1) and noncoding genes (C2), moderate confidence testis‐specific expressed protein‐coding genes (C3) and noncoding genes (C4), low‐confidence testis‐specific expressed genes (C5), genes with transcripts exhibiting a testis‐specific expression pattern (C6a), and genes without transcripts exhibiting a testis‐specific expression pattern (C6b). In this study, only 1061 protein‐coding genes and 1184 long noncoding RNAs (lncRNAs) exhibited testis‐specific expression pattern with high confidence (C1 and C2 groups) as well as a median expression greater than 0.5 fragments per kilobase of exon model per million reads mapped (FPKM) in normal testis samples were included.^6^ According to GENCODE v19 annotation data, lncRNAs were reclassified into 6 biotypes,^17^ including three prime overlapping ncRNA (lncRNAs located within the 3' UTR of protein‐coding genes), antisense (lncRNAs overlapping any protein‐coding genes on the opposite strand), lincRNA (long intergenic noncoding RNA with a length greater than 200 bp), sense intronic (lncRNAs located within the intron of any protein‐coding gene), sense overlapping (lncRNAs within any protein‐coding gene within its intron on the coding strand), and processed transcript (transcripts without an open reading frame). Testis‐specific expressed genes exhibited expression (normalized read counts >5) in at least 1% TGCT samples were defined as expressed.

### 2.2 Differential gene expression analysis

To further explore the expression difference of TSGs in malignant (TGCT) and normal testis samples, we performed differential expression analysis by using data from The Cancer Genome Atlas (TCGA) project (https://xenabrowser.net/datapages/) and the GTEx portal. Expression data of 174 normal testes were obtained from GTEx Analysis V7 (dbGap Accession phs000424.v7.p2, https://www.gtexportal.org/home/datasets) and quantified as raw read counts. Differential expression analysis was performed with R package DESeq2. The batch effect was removed by sva package and raw read counts data was normalized by DESeq2. The magnitude (log2 transformed fold change) and significance (*P‐*value) of differential expression between groups were calculated, and genes with |log2foldchange|≥1 and Benjamini–Hochberg false discovery rate (FDR) adjusted *P *< 0.05 were considered as significant.

### 2.3 Pathway enrichment analysis

To investigate the potential function of altered TSGs in TGCT samples, we performed pathway enrichment analysis based on GO Biological Process Ontology gene sets with the R Bioconductor package clusterProfiler. As most lncRNAs were not included in the pathway annotation data, only protein‐coding genes were included in this analysis. TSGs with significantly altered expression in TGCT samples from that in normal testis tissues were included in this analysis (|log2foldchange|≥1 and *P*_fdr_ < 0.05). Pathway with enrichment *P* < 0.01 was considered as significant.

### 2.4 Survival analysis

The clinical and follow‐up information of 134 TGCT samples were obtained from a previous TCGA study^18^ (Table S1). As for the few disease‐specific survival and overall deaths of TGCT patients, we used disease‐free interval (DFI) and progression‐free interval (PFI) as clinical outcome endpoints.^18^ We performed a multivariate Cox proportional hazards regression model to calculate the Hazard Ratio (HR), the 95% confidence interval (95% CI), and *P*‐values with adjustments for age and tumor stage. The Kaplan‐Meier (K‐M) method was used to create the survival plots and the log‐rank test was used to compare the difference in survival curves.

### 2.5 Statistical analysis

Wilcoxon rank‐sum test was performed to compare the number of expressed CT genes in different groups. Spearman rank‐sum test was used to evaluate the association between the expression level of CT genes and the stemness scores, the fraction of altered genomes, and immune cells level in 133 samples. The stemness scores, the fraction of altered genomes and immune cells information were obtained from a previous study.^19^ General statistical analyses were performed with R software (R version 3.2.2).

## 3 RESULTS

### 3.1 Characterization of cancer‐testis genes in testicular germ cell tumor

**Figure 1:**
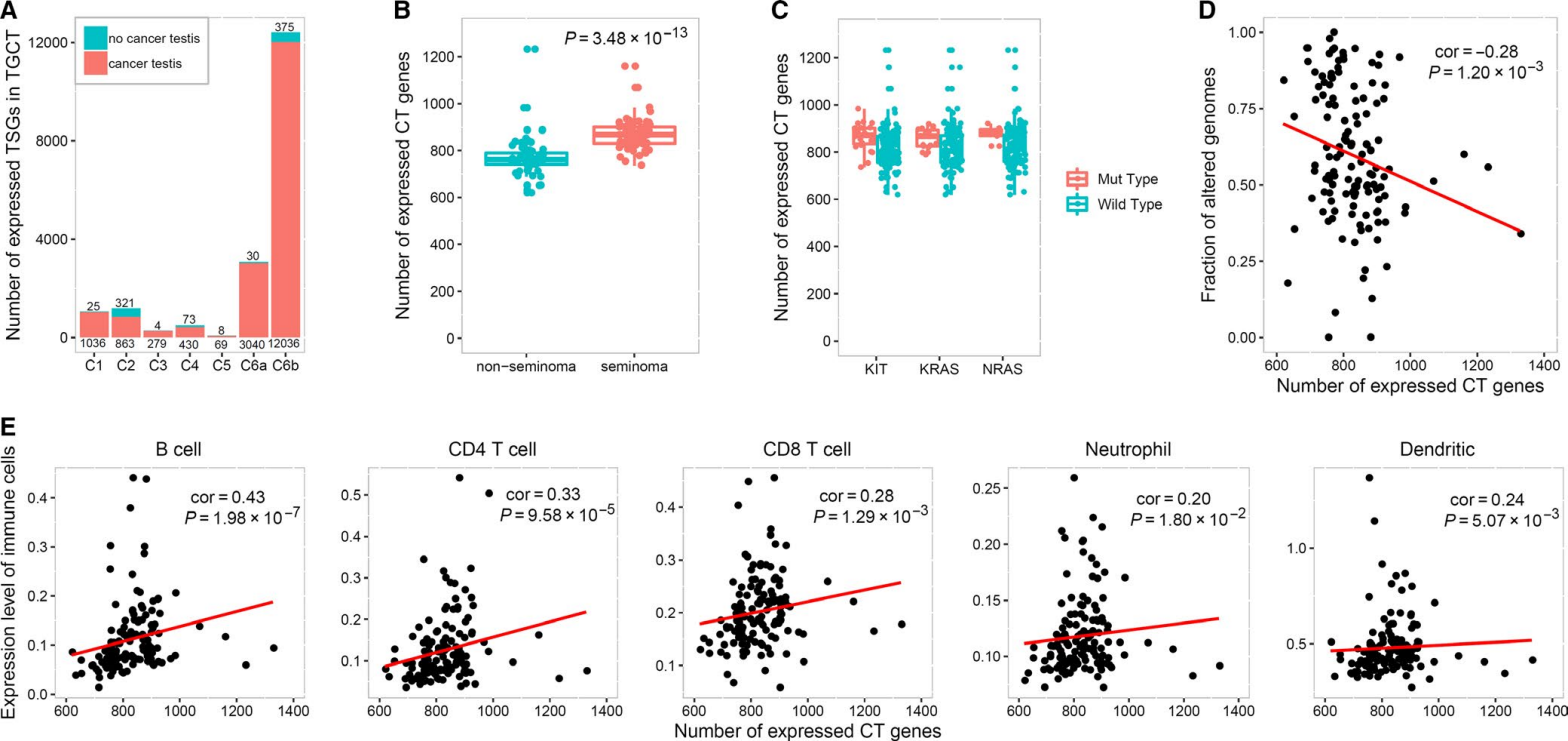
Classification of CT genes and the association with patient characteristics. A, The number of expressed testis‐specific expressed genes and CT genes. B, The number of expressed CT genes in 2 histologic subtypes. C, Association of the number of expressed CT genes and 3 significantly mutated genes of TGCT. D, Association of the number of expressed CT genes and fraction of copy number altered genomes in TGCT samples. E, Association of the number of expressed CT genes and expression level of immune cells in TGCT samples

We first investigated the expression pattern of TSGs in 150 TGCT samples from the TCGA project. In total, 97.64% (1036/1061) of our previously defined testis‐specific expressed protein‐coding genes (C1 group) and 72.89% (863/1184) testis‐specific expressed noncoding RNAs (C2 group) were expressed (normalized read counts >5) in at least 1% TGCT samples (Figure 1A, Table S2), including 883 CT genes and 710 CT‐lncRNAs defined in other 19 cancer types^6^, ^20^ (Table S2).

In an attempt to explore the relationship between CT genes and clinical characteristics in TGCT samples, we performed subgroup analysis and identified that the number of expressed CT genes was significantly higher in the seminoma histologic subtype than other non‐seminoma subtypes (Median_seminomas_ = 867.5, Median_non‐seminomas_ = 764; *P *= 3.48 × 10^−13^) (Figure 1B). Further differential expression analysis between 68 seminomas and 65 nonseminomas revealed that 366 TSGs showed elevated expression in the seminomas and 165 were decreased. Interestingly, although spermatogenesis‐related genes shared a similar expression level in both two groups of patients, meiotic cell cycle‐related genes were specifically elevated in the seminoma type (Table S3‐S4). Additionally, we identified that CT genes were more prone to be expressed in samples with mutations in the only 3 significantly mutated genes identified previously,^10^ including *KIT* (Median_Mut‐type(MT)_ = 871, Median_Wide‐type(WT)_ = 804, *P* = 9.18 × 10^−4^), *KRAS* (Median_MT_ = 867, Median_WT_ = 806, *P* = 1.49 × 10^−2^), and *NRAS* (Median_MT_ = 876, Median_WT_ = 820, *P* = 3.99 × 10^−2^) (Figure 1C). However, the number of expressed CT genes showed a negative moderate correlation with the fraction of copy number altered genomes (cor = −0.28, *P* = 1.20 × 10^−3^) (Figure 1D).

As CT genes were commonly considered as immunotherapy targets, we further evaluated the association of expressed CT genes with the estimated expression of 6 types of immune cells (B cells, CD4 T cells, CD8 T cells, neutrophils, macrophages and dendritic cells) in 133 TGCT samples. Interestingly, we identified moderate associations between the number of expressed CT genes and the expression level of 5 types of immune cells, including B cells (cor = 0.43 *P* = 1.98 × 10^−7^), CD4 T cells (cor = 0.33, *P* = 9.58 × 10^−5^), CD8 T cells (cor = 0.28, *P* = 1.29 × 10^−3^), neutrophils (cor = 0.20, *P* = 1.80 × 10^−2^), and dendritic cells (cor = 0.24, *P* = 5.07 × 10^−3^) (Figure 1E). Additionally, further co‐expression analysis revealed that the number of expressed CT genes were also significantly associated with *PD‐1*/*PD‐L1* gene expression (*PD‐1*: cor = 0.47, *P* = 1.10 × 10^−8^; *PD‐L1*: cor = 0.23, *P* = 8.58 × 10^−3^; Figure S1).

### 3.2 A comprehensive comparison of cancer‐testis genes in malignant and normal testis samples

**Figure 2:**
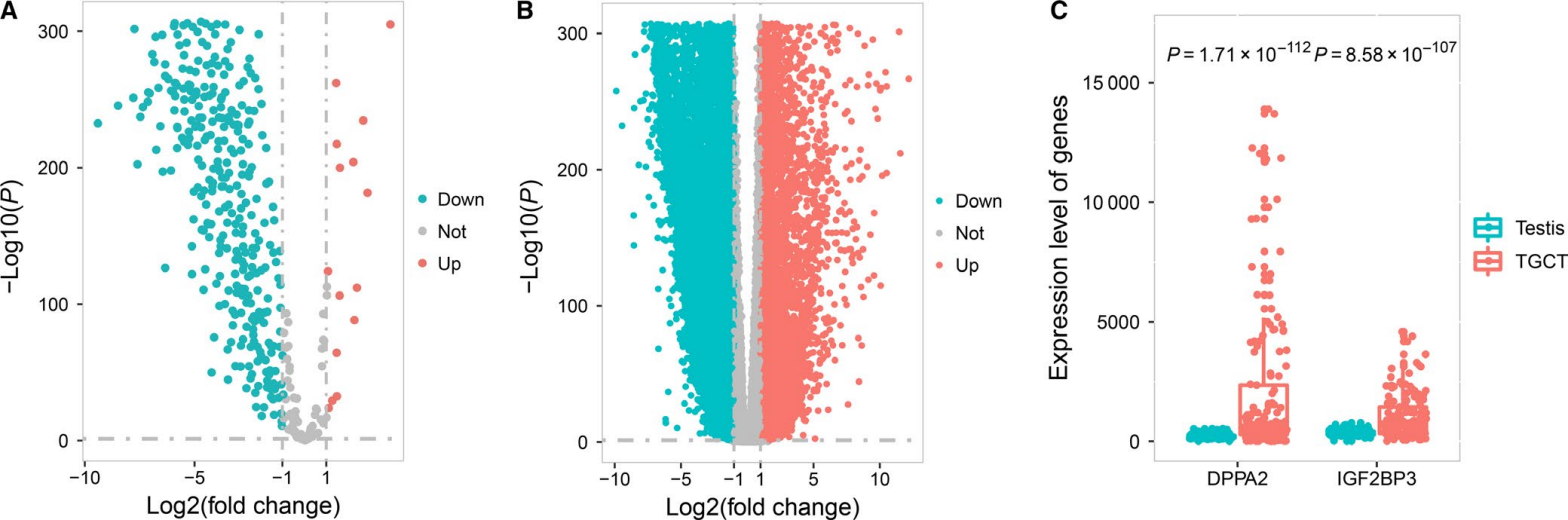
A, Differential expression analysis of testis‐specific expressed genes. Volcano plot displayed differentially expressed TSGs in TGCT and normal testis samples. B, Differential expression analysis of genes without testis‐specific expression pattern. Volcano plot displayed differential expressed genes without testis‐specific expression pattern in TGCT and normal testis samples. C, Differential expression analysis of testis‐specific expressed genes. Differential expression pattern of *DPPA2* and *IGF2BP3* in TGCT and normal testis samples

As TSGs were highly expressed in both malignant (TGCT) and normal testis samples, we then evaluated the differential expression of TSGs in 174 normal testis samples and 150 TGCT samples. Interestingly, we identified that 96.16% (1826/1899) of the TSGs genes were differentially expressed between malignant and normal testis samples (Figure 2A, Table S5) while 53.48% (6637/12411) of the genes without testis‐specific expression pattern were differentially expressed (Figure 2B). Additionally, as the majority of the differential expressed TSGs (98.69%, 1802/1826) were down‐regulated in TGCT patients (Figure 2A), only 54.26% (3601/6637) of the differential expressed genes without testis‐specific expression pattern were down‐regulated in TGCT (Figure 2B).

Of the 1802 down‐regulated TSGs in TGCT samples, 951 were protein‐coding genes, and 851 were lncRNAs, including 125 classic CT genes, such as the MAGE family (*MAGEA1*: log2FC = −4.04, *P* = 1.65 × 10^−115^; *MAGEA3*: log2FC = −3.18, *P* = 3.75 × 10^−55^; *MAGEA4*: log2FC = −1.98, *P* = 1.64 × 10^−18^; *MAGEA8*: log2FC = −1.74, *P* = 1.30 × 10^−39^; *MAGEA10*: log2FC = −2.56, *P* = 4.87 × 10^−55^; *MAGEA11*: log2FC = −3.63, *P* = 4.28 × 10^−193^ and *MAGEA12*: log2FC = −2.24, *P* = 3.34 × 10^−25^) and HORMAD family (*HORMAD1*: log2FC = −3.45, *P* = 6.84 × 10^−185^ and *HORMAD2*: log2FC = −7.43, *P < *2.20 × 10^−16^). The 24 up‐regulated TSGs including 22 protein‐coding genes and 2 lncRNAs, of which 2 were classic CT genes (*DPPA2*: log2FC = 2.35, *P* = 1.71 × 10^−112^ and *IGF2BP3*: log2FC = 1.57, *P* = 8.58 × 10^−107^) (Figure 2C, Table S5).

### 3.3 Stem cell maintenance genes are up‐regulated in testicular germ cell cancer

**Figure 3:**
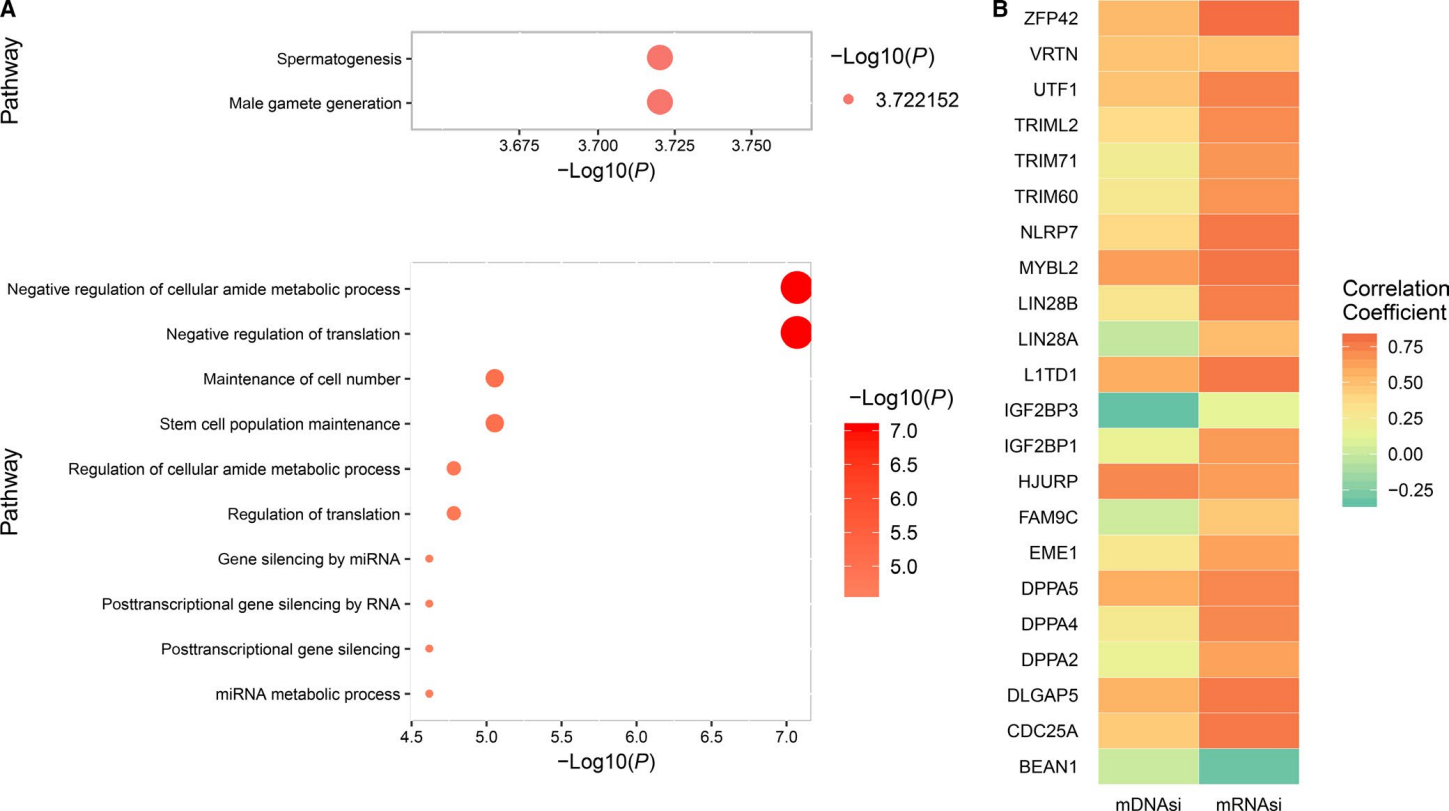
Pathway enrichment analysis of differentially expressed testis‐specific expressed genes. A, Dotplot depicts the enriched pathways of differentially expressed testis‐specific expressed protein‐coding genes in TGCT samples compared with normal testis samples. B, Heatmap depicts the correlation of testis‐specific expressed protein‐coding genes in stem cell maintenance related pathways with the stemness signatures

To evaluate the potential function of differential expressed testis‐specific protein‐coding genes, we performed pathway enrichment analysis with GO Biological Process Ontology dataset. The result revealed that the down‐regulated TSGs in TGCT were significantly enriched in 2 pathways, including spermatogenesis (*P* = 1.90 × 10^−4^) and male gamete generation (*P* = 1.90 × 10^−4^) pathways (Figure 3A, Table S6); while the up‐regulated testis‐specific protein‐coding genes in TGCT samples were involved in translational regulation and stem cell maintenance related pathways, such as negative regulation of translation (*P* = 9.04 × 10^−8^), regulation of translation (*P* = 1.07 × 10^−5^), stem cell population maintenance (*P* = 9.11 × 10^−6^), somatic stem cell population maintenance (*P* = 8.88 × 10^−4^), and stem cell proliferation (*P* = 8.88 × 10^−4^) (Figure 3A, Table S7). Interestingly, although the number of expressed CT genes was significantly higher in the seminoma group with *KIT*, *KRAS* and *NRAS* mutations, expression of 4 genes in stem cell maintenance related pathways were significantly elevated in the nonseminoma group and samples that without driver gene mutations (Figure S2).

As 6 of 9 stem cell maintenance related pathways included in GO Biological Process Ontology dataset were over‐represented in testis‐specific expressed protein‐coding genes, we further evaluated the association of 22 up‐regulated testis‐specific expressed protein‐coding genes with the stemness scores. Interestingly, 16 of the 22 up‐regulated testis‐specific expressed protein‐coding genes showed strong positive associations with both epigenetic‐related and expression‐related stemness signatures (Cor ≥ 0.2, *P*_adj_ < 0.05) (Figure 3B, Table S8).

### 3.4 Survival outcome analysis

**Figure 4:**
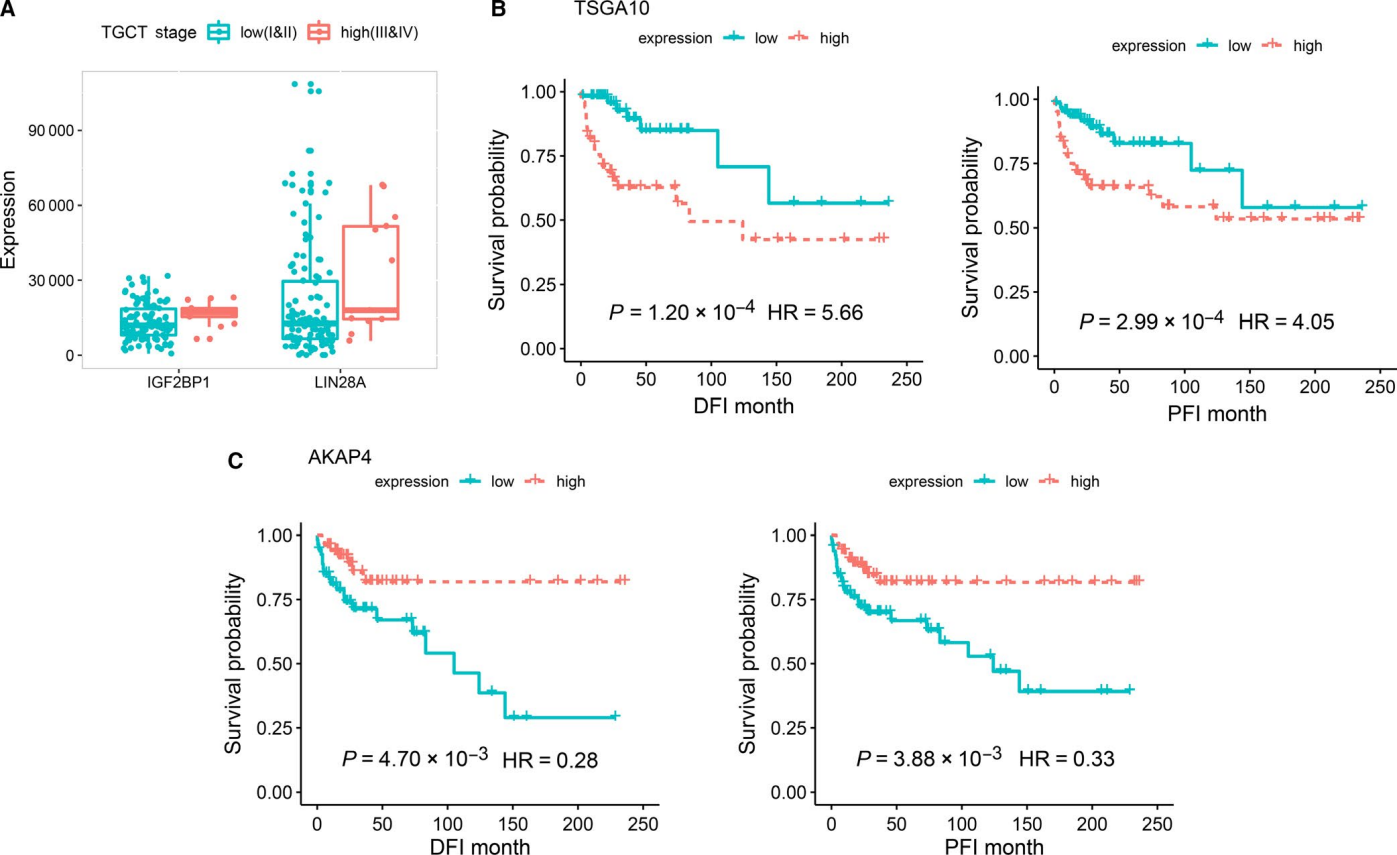
Survival analysis of testis‐specific expressed genes. A, Association of testis‐specific expressed genes with clinical tumor stages. B‐C, Kaplan‐Meier plot depicts the association of testis‐specific expressed genes with TGCT prognosis statuses

To evaluate the prognostic effect of TSGs in TGCT patients, we performed survival analysis with DFI and PFI as the clinical endpoints as recommended in the previous study.^10^ In this analysis, we used 30 days past diagnosis as a duration of the primary treatment interval. The baseline and follow‐up information of the patients were shown in Table S1. The median DFI event time was 27.03 months, and the median PFI event time was 28.18 months. Wilcoxon rank‐sum analysis revealed that four TSGs exhibited significantly higher expression in high‐stage (III, IV) TGCT patients, while 59 TSGs showed higher expression level in low‐stage (I, II) TGCT patients (*P *< 0.01). Of the 59 TSGs that showed negative correlations with TGCT progression, 45 were protein‐coding genes, including 6 classic CT genes (*PIWIL2*: median_high_ = 619.45, median_low_ = 1425.66, *P* = 7.79 × 10^−3^; *CCDC36*: median_high_ = 28.89, median_low_ = 59.83, *P* = 8.37 × 10^−3^; *TFDP3*: median_high_ = 1.26, median_low_ = 22.92, *P* = 4.24 × 10^−3^; *FBXO39*: median_high_ = 7.26, median_low_ = 19.30, *P* = 2.64 × 10^−3^; *CXorf48*: median_high_ = 2.14, median_low_ = 5.07, *P* = 7.96 × 10^−3^ and *HEMGN*: median_high_ = 0.00, median_low_ = 2.13, *P* = 1.06 × 10^−3^). Interestingly, 2 of the stem cell population maintenance related genes showed a positive correlation with the progression of TGCT samples, including *LIN28A* (median_high_ = 18045.24, median_low_ = 12731.15, *P* = 0.03) and *IGF2BP1* (median_high_ = 17224.03, median_low_ = 12036.74, *P* = 0.03) (Figure 4A, Table S9).

With the multivariate Cox proportional hazards regression model, we identified a total of 48 TSGs that were associated with DFI, 38 were associated with PFI and 20 were consistently associated with both clinical ends (*P *< 0.01) (Table S10). Of the 20 survival‐related TSGs, 2 were previously reported CT genes, including *TSGA10* and *AKAP4*. TGCT patients with higher expression of *TSGA10* had a significantly worse survival than those with low expression when using both PFI (HR = 4.05, *P* = 2.99 × 10^−4^) and DFI (HR = 5.66, *P* = 1.20 × 10^−4^) as clinical endpoints after controlling for age and tumor stage (Figure 4B, Table S10). However, an inverse association was observed for *AKAP4* as higher expression of this gene was associated with a better survival of TGCT (PFI: HR = 0.33, *P* = 3.88 × 10^−3^; DFI: HR = 0.28, *P* = 4.70 × 10^−3^) (Figure 4C, Table S10). Interestingly, the stem cell maintenance related gene *LIN28A* was also associated with a better survival of TGCT (PFI: HR = 0.36, *P* = 5.81 × 10^−3^; DFI: HR = 0.32, *P* = 6.64 × 10^−3^) (Table S10).

## 4 DISCUSSION

CT genes are a class of genes predominantly expressed in the germ cells of testis and are ectopically reactivated in various types of tumor cells. Accumulating evidence suggested that this group of genes can also serve as driver candidates in the development of cancer.^3^ However, previous studies focused mainly on nongerm cell tumors although some classic CT genes have also been found in testicular germ cell tumors.^11^, ^12^ Interestingly, TGCT is a kind of cancer characterized by genomic de‐methylation and a low genomic alteration rate,^10^ which indicates the important role of transcriptome alteration during tumorigenesis. Therefore, systematically investigation of CT genes in TGCT could unravel the role of this group of genes in the development of TGCT. In this study, we performed a comprehensive description of CT genes in TGCT and provided the first evidence that CT genes play important roles in the progression and maintenance of TGCT.

Of the two most common types of TGCT, although seminomas are less aggressive,^8^ we observed a significant enrichment of expressed CT genes in this type of cancer. Interestingly, consistent with our previous hypothesis that de‐methylation was an important mechanism for the reactivation of CT genes in cancers,^6^ seminomas are also a group of cancers that lack of DNA methylation.^10^ Thus, the globally de‐methylated genomes of seminoma cells could serve as the epigenetic mechanism for CT genes expression in TGCT.^6^ However, in contrast to non‐germ cell solid cancer,^6^ CT genes in TGCT are prone to be expressed in samples with driver genes (*KIT*, *KRAS* and *NRAS*) mutations, which may be attributed to the altered aneuploidy and genomic instability in these samples. Additionally, we also observed a positive association between the number of expressed CT genes with different types of immune cells. Recently, there is accumulating evidence confirming the association between immune biomarkers and the response to anti‐PD‐1/L1 therapy across several solid tumors.^21^, ^22^ In this study, we found that *PD‐1*/*PD‐L1* expression was positively correlated with the number of expressed CT genes, suggesting that CT genes represent a potential marker for effective PD‐1/L1 immunotherapy.

During the tumorigenesis of the testis, we identified that the expression level of most CT genes in spermatogenesis pathway was de‐regulated, while the expression of genes in the maintenance of stem cells was up‐regulated. As TGCT is a cancer type derived from immature germ cells that retain expression of pluripotency genes,^9^ further correlation analysis provided additional evidence that CT genes up‐regulated in TGCT samples showed strong associations with the stemness scores. Stemness is defined as the ability of self‐renewal and differentiation for normal cells, as well as the shared characteristic with cancer cells.^23^ Recently, increasing evidence suggested that the development of cancer cells involves the acquisition of stem‐cell‐like phenotypes and loss of differential features, which may lead to the progression and distant metastasis of cancer cells.^23^, ^24^, ^25^ Interestingly, we identified that *LIN28A* and *IGF2BP1*, the essential members in stem cell maintenance, were also associated with advanced tumor stages in addition to the oncogenic effect in tumorigenesis.^26^, ^27^ Thus, we proposed that the dysregulation of stemness‐related CT genes in TGCT may not only lead to the initiation of tumorigenesis and oncogenic de‐differentiation, but also contribute to the progression of testicular germ cells.

Consistent with our hypothesis, further survival analysis revealed that 22 genes were associated with the prognosis of TGCT, including 2 previously known CT genes. *TSGA10*, with the highest expression level in elongating spermatids and localized in the fibrous sheath of mature sperm, has previously been reported to be a biomarker in brain tumors^28^, ^29^ and regulated the development of esophageal squamous cell carcinoma.^30^ *AKAP*4, as a kinase anchor protein, has been reported to contribute to the development of cervical cancer and ovarian serous carcinoma.^31^, ^32^ In this current study, we first provided evidence for the prognostic effect of *TSGA10* and *AKAP4* in the progression of TGCT, which may serve as potential treatment targets. Additionally, we identified that although some CT genes have previously been reported to be oncogenic, they could also predict a better survival with either DFI or PFI as the endpoint. *LIN28A* is a highly conserved RNA binding protein and has emerged as an oncogenic driver by blockading of let‐7 micro‐RNA biogenesis in many cancers^33^, ^34^, ^35^; however, in this study, we identified that *LIN28A* was associated with a better prognosis of TGCT, which is different from the other cancers types.^36^, ^37^, ^38^ Nevertheless, there also exists some limitations in this study. As for the small sample size used in this study as well as no independent validations, further studies are warranted to validate the findings.

In summary, we identified a total of 1899 testis‐specific expressed genes (1036 testis‐specific protein‐coding genes and 863 testis‐specific long noncoding genes), including 1593 CT genes (883 CT genes and 710 CT‐lncRNAs) in TCGA TGCT samples. By integrating expression from both normal and malignant testis samples, we proposed that CT genes up‐regulated in TGCT were primarily involved in stem cell maintenance and could serve as prognostic factors. This founding greatly broadens our understanding of CT genes in testicular germ‐cell tumors.

## CONFLICT OF INTEREST

The authors declare no conflict of interest.

## Data Availability

Data sharing is not applicable to this article as no new data were created or analyzed in this study.
