# Supplementary material for: Comprehensive characterization of cancer‐testis genes in testicular germ cell tumor
Source: Cancer Med. 2019 May 9;8(7):3511–9. doi: 10.1002/cam4.2223 (PMC6601584; doi:10.1002/cam4.2223)
Supplement: Supplementary file 1 [file CAM4-8-3511-s001.docx]

**Figure S1. Correlation between the number of expressed CT genes and expression level of *PD-1*/*PD-L1*.**

**Figure S2. Expression of four genes in seminoma and non-seminoma groups.**
